# Understanding the Relationships between Free Asparagine in Grain and Other Traits to Breed Low-Asparagine Wheat

**DOI:** 10.3390/plants11050669

**Published:** 2022-02-28

**Authors:** Joseph Oddy, Sarah Raffan, Mark D. Wilkinson, J. Stephen Elmore, Nigel G. Halford

**Affiliations:** 1 Plant Sciences Department, Rothamsted Research, Harpenden AL5 2JQ, UK; joe.oddy@rothamsted.ac.uk (J.O.); sarah.raffan@rothamsted.ac.uk (S.R.); mark.wilkinson@rothamsted.ac.uk (M.D.W.); 2 Department of Food and Nutritional Sciences, University of Reading, Whiteknights, P.O. Box 226, Reading RG6 6AP, UK; j.s.elmore@reading.ac.uk

**Keywords:** wheat, asparagine, breeding, acrylamide, protein, pre-harvest sprouting, nitrogen-use efficiency, senescence

## Abstract

Since the discovery of acrylamide in food, and the identification of free asparagine as the key determinant of acrylamide concentration in wheat products, our understanding of how grain asparagine content is regulated has improved greatly. However, the targeted reduction in grain asparagine content has not been widely implemented in breeding programmes so far. Here we summarise how free asparagine concentration relates to other quality and agronomic traits and show that these relationships are unlikely to pose major issues for the breeding of low-asparagine wheat. We also outline the strategies that are possible for the breeding of low-asparagine wheat, using both natural and induced variation.

## 1. Introduction

Wheat is one of the world’s most important crops, contributing an estimated 18.6% to global daily calorie intake and 19.8% to global daily protein intake in 2018 [1]. The contribution of wheat to daily calorie and protein intake varies substantially by region though, with certain regions having greater dependence on wheat than others. For example, the contribution of wheat to daily calorie and protein intake was approximately double the global average at 39.1% and 38.4%, respectively, in Central Asia in 2018 (Kazakhstan, Kyrgyzstan, Tajikistan, Turkmenistan, and Uzbekistan (as described by the Food and Agriculture Organisation of the United Nations) [1]). Consequently, it is essential to ensure that the supply and quality of wheat is safeguarded against emerging challenges. This can be achieved by the development of new crop management strategies and crop protection products, or through the breeding of new varieties.

Wheat breeding and research has been greatly facilitated in recent years by the sequencing of multiple wheat genomes [2], the development of numerous marker technologies [3], and the use of new gene editing technologies [4]. With the development of these technologies, we can begin to investigate and improve traits that may have been prohibitively costly or time consuming to improve in the past, and the free amino acid composition of wheat grain is one such trait.

The free (soluble, non-protein) amino acid content of wheat grain has been of most interest to wheat geneticists in recent years because of the food safety issues associated with free asparagine, the precursor to the ‘probably carcinogenic’ processing contaminant, acrylamide [5]. Free asparagine reacts with reducing sugars to form acrylamide [6,7], but free asparagine concentration has been shown to be the major determinant of acrylamide concentration in wheat products in several studies (see [8] for review). Halford et al. [9], for example, used data generated by Muttucumaru et al. [10], to plot free asparagine concentration against acrylamide formation in wheat flour heated for 20 min at 160 or 180 °C, and obtained coefficients of determination (R^2^) of 0.956 and 0.998 for pot- and field-grown plants, respectively. In contrast, there was no relationship between the concentration of reducing sugars and the amount of acrylamide that formed. Muttucumaru et al. had shown sulphur deficiency to cause very high concentrations of free asparagine to accumulate in wheat grain, and Granvogl et al. [11] obtained very similar results, with acrylamide formation closely related to asparagine concentration, except in flours from extremely sulphur-deprived plants, in which free asparagine concentration was so high that it was no longer limiting.

**Figure 1:**
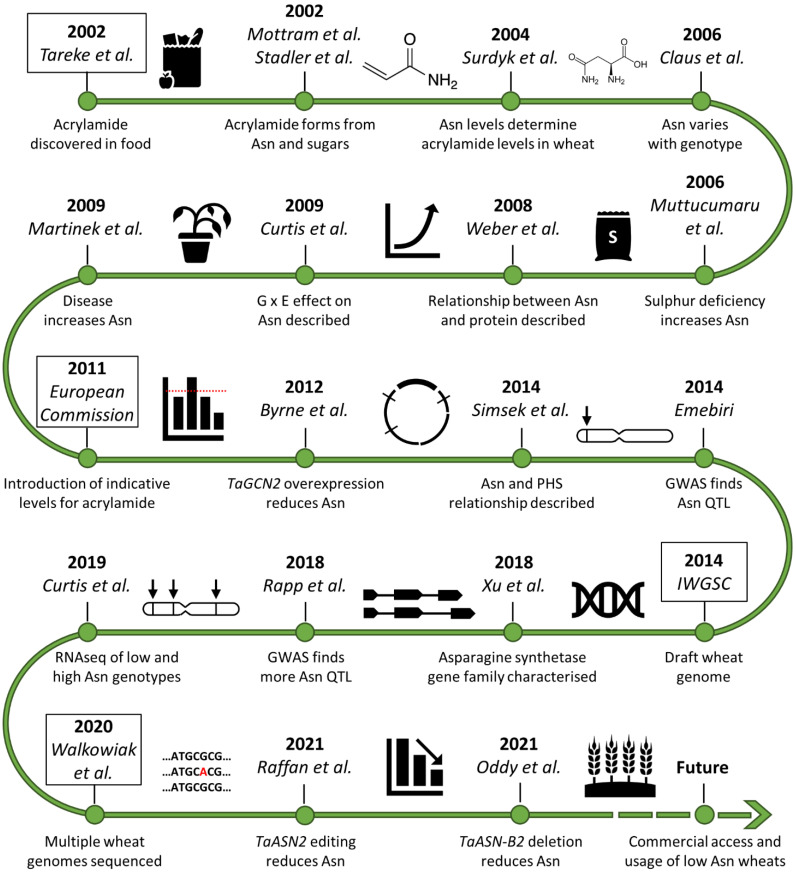
Timeline of asparagine research in wheat since the discovery of acrylamide in food [2,5,6,7,10,12,13,14,15,16,17,18,19,20,21,22,23,24,25,26]. Asn (asparagine), G × E (genotype-by-environment interaction), PHS (pre-harvest sprouting), GWAS (genome-wide association study), QTL (quantitative trait locus/loci), RNAseq (RNA sequencing).

Curtis et al. [15] also studied the effect of sulphur deficiency and showed acrylamide formation to rise with free asparagine concentration (R^2^ = 0.9945), up to free asparagine concentrations of approximately 25 mmol/kg or higher, something only seen in flour from extremely sulphur-deprived plants.

Acrylamide also forms in potato products and the relationship between free asparagine and reducing sugar concentration and acrylamide formation for potato is very different. Potato tubers have higher concentrations of free asparagine than cereal grains and reducing sugar concentration is usually the limiting factor for acrylamide formation, although free asparagine concentration does contribute to the variance in some datasets [27]. Nevertheless, the clear relationship between free asparagine concentration and acrylamide formation in wheat products means that strategies to control acrylamide formation in wheat-based foods over the last 20 years have targeted free asparagine (Figure 1).

Although we now better understand the environmental and genetic factors that influence grain asparagine content, there are still many unanswered questions around how these factors interact and how they relate to other traits. Here, we summarise some of the research regarding the relationship between free asparagine concentration in the grain and other traits, and how genetic improvements might be made using this information.

## 2. Relationships between Free Asparagine, Quality and Agronomic Traits

### 2.1. Free Asparagine Concentration and Quality Traits

Quality traits in wheat are those that impact the functionality of the end product (i.e., the baking and nutritional quality of the grain), so encompass traits such as pre-harvest sprouting (PHS), protein content and hardness. Grain-free asparagine content has sometimes been found to correlate with some of these quality traits, but this differs greatly between studies (Table 1 and Table 2). Few quality traits have been tested for a relationship with free asparagine in more than one study, and those that have often show different relationships across studies (Table 1), implying that free asparagine concentration is unlikely to correlate strongly with quality traits. 

Malunga et al. [28] undertook the largest study of free asparagine in relation to quality traits, screening 42 quality traits and assessing their relationship with free asparagine, in both wholemeal and white flours. This analysis revealed that free asparagine in wholemeal samples did not correlate with any quality parameters, except for a weak correlation (*r* = −0.389, *p* = 0.0339) with the extensograph A parameter. Similarly, free asparagine in white flour only correlated weakly with extensograph *R_max_* (*r* = −0.370, *p* = 0.0444), extensograph *A* (*r* = −0.378, *p* = 0.0394) and water dough colour b* parameters (*r* = 0.373, *p* = 0.0426). Corol et al. [29] also performed correlation analyses of free asparagine with quality traits and did find some weak associations, but these have not been corroborated by further studies (Table 1). 

One potentially interesting relationship is that between free asparagine and PHS, because of the potential for protein hydrolysis during PHS to release free asparagine. PHS negatively impacts wheat quality in a range of ways, reducing flour yield, the quality of baked products, and nutrient content [30]. Simsek et al. [20] reported a moderately strong (*r* = 0.6–0.7) positive correlation between free asparagine, sprouting score, and endoprotease activity in samples of sprouted wheat grain, suggesting that there was a relationship between asparagine and PHS at high levels of sprouting. Additionally, in a study designed to render the asparagine synthetase 2 genes (*TaASN2*) non-functional through gene editing, Raffan et al. [25] observed a poor germination phenotype that could be rescued through exogenous application of asparagine to the soil, implying that low-grain asparagine content may inhibit germination and could perhaps also affect PHS. Further research is required to confirm the germination phenotype, but asparagine synthetases are known to play important roles in germination in other species [31,32]. No correlation has been observed to date between asparagine and Hagberg falling number (HFN) (Table 1), which is indicative of α-amylase activity and, therefore, PHS. However, it is possible that a relationship between grain asparagine content, germination and PHS could exist when asparagine concentration is very low (e.g., in *TaASN2* edited lines) or very high (e.g., in artificially sprouted wheat samples).

**Table 1:** Association between free asparagine and selected quality traits.

Asn Measurement	Trait	*r*	*p*	Reference
Log*_e_* transformation	Farinograph absorption	0.94	<0.001	[33]
	Nitrogen: sulphur grain content	0.73	<0.01	
	Nitrogen grain content	0.62	<0.05	
Log*_e_* transformation	Sprouting score	0.68	<0.001	[19]
	Endoprotease activity (sprouted)	0.69	<0.001	
	Endoprotease activity (ΔD)	0.60	<0.01	
Untransformed	HFN	0.07	0.39	[29]
	Z-SDS	0.37	<0.001	
	Gluten content	0.44	<0.001	
	Starch content	−0.32	<0.001	
	Water absorption	0.35	<0.001	
	Hardness index	0.03	0.68	
Log*_e_* transformation	Absorption	−0.03	>0.05	[34]
Untransformed	Hardness index	0.15	>0.05	[35]
Log_10_ back-transformed	Sulphur grain content	0.14	>0.05	[23]
	HFN	0.03	>0.05	
	Z-SDS	−0.29	<0.001	
Untransformed	HFN	−0.17	0.36	[28]
	Gluten index	−0.36	<0.05	
	Flour starch damage	−0.18	0.33	
	Farinograph absorption	−0.12	0.5436	

Asn (asparagine), HFN (Hagberg falling number), Z-SDS (Zeleny sedimentation index).

In contrast to other quality traits, the relationship between grain asparagine content and protein has been tested numerous times and the results suggest that there is a positive correlation between the two traits, varying from weak to strong, under different conditions (Table 2). The protein content of wheat is important both for breadmaking functionality and for its nutritional quality, especially as the global agricultural system shifts towards the cultivation of more plant protein for sustainability reasons. A more detailed analysis of the relationship between protein and asparagine was undertaken by Simsek et al. [19], who found significant positive associations between asparagine and extractable F4 (albumin/globulin), F5, and F6 (hydrolysed polymeric/non-gluten protein) HPLC protein fractions. This is consistent with the release of free asparagine from the hydrolysis of proteins under PHS. Simsek et al. [19] also found significant negative associations between asparagine and unextractable F1 (HMW glutenin polymers) and F2 (LMW glutenin polymers) protein fractions. This was further supported by Ohm et al. [36], where significant negative (*p* < 0.05) genotypic and phenotypic correlations were found between free asparagine and unextractable F1 protein fractions, but not between free asparagine and extractable F1 fractions.

The contrasting relationships between free asparagine and the different protein fractions has interesting implications for quality, because the ratio of unextractable HMW polymeric proteins to extractable LMW polymeric proteins is a better determinant of quality than total protein measurements [37,38]. Consequently, lower free asparagine content in the grain may be associated with higher bread-making quality. This conclusion was drawn by Ohm et al. [36], who further suggested that measurements of unextractable polymeric protein may allow for selection of varieties that simultaneously have high-quality bread-making potential and are low in free asparagine content. Such a correlation between free asparagine and bread-making quality has not been consistently observed across studies, though (see Table 1), so the relationship is probably more complex than this. Higher protein content may also be desirable, independent of its effect on bread-making quality.

The complexity of the factors determining the free asparagine content of grain and protein can be illustrated by looking at soft wheat varieties. These varieties typically have lower protein content than hard wheats, making them unsuitable for bread-making but suitable for biscuits, breakfast cereals, pastries and other baked goods. Based on this, the grain of soft wheats might be expected to have lower free asparagine content than hard wheats, due to the positive correlations often found with protein (Table 2). Curtis et al. [39] did show that varieties with consistently low free asparagine concentration were often soft wheats, but the difference between hard and soft variety groups was not significant, with high and low-asparagine varieties in both groups. It is possible that the association of some soft wheat varieties with consistently low free asparagine content was due to the deletion of one of the asparagine synthetase 2 homeologues, *TaASN-B2*, which has been shown to be associated with lower grain asparagine content and was more common in the soft wheats used in the trial [26]. The effect of this deletion is only apparent when the plants have adequate sulphur, though, with the effect being overwhelmed by the huge increase in free asparagine concentration that occurs under sulphur deficiency [26], adding more complexity to the control of grain asparagine content.

**Table 2:** Associations between free asparagine and protein content.

Asparagine Measure	Protein Measure	R^2^*/r*	*p*	Reference
Untransformed	Crude protein	0.86 *	<0.001	[14]
Untransformed	Protein content (2006 UN)	0.93	<0.01	[16]
	Protein content (2006 T)	0.63	<0.05	
	Protein content (2007 UN)	0.75	>0.05	
	Protein content (2007 T)	0.27	>0.05	
	Protein content (2006 N)	0.73	<0.01	
	Protein content (2007 N)	0.89	<0.01	
Log*_e_* transformation	Protein content (non-sprouted)	NA	>0.05	[19]
	Protein content (sprouted)	NA	>0.05	
	Protein content (ΔD)	NA	>0.05	
Untransformed	Total protein content	0.45	<0.001	[29]
	Wholemeal protein content	0.51	<0.001	
	Flour protein content	0.38	<0.001	
Log*_e_* transformation	Protein content	0.43	<0.001	[34]
Log*_e_* transformation	Protein content (*r_p_*)	−0.03	>0.05	[36]
	Protein content (*r_g_*)	−0.37	>0.05	
Untransformed	Total protein content	0.52	<0.01	[35]
Log_10_ back transformed	Total protein content	0.23	<0.01	[23]
Untransformed	Crude protein	0.36 *	NA	[40]
Untransformed	Crude protein	0.04 *	NA	[41]
Untransformed	Wholemeal protein content	−0.08	0.66	[28]
	Flour protein content	−0.14	0.46	

* These values refer to R^2^ values, not *r* values. *r_p_* (phenotypic correlation), *r_g_* (genotypic correlation).

Although the relationship between free asparagine content and the protein composition of grain is complex, there are two factors that are well known to affect both: nitrogen and sulphur fertilisers. Nitrogen application increases both the free asparagine and protein content of grain, whereas sulphur application decreases free asparagine content and improves protein composition (see [42] for review). This is reflected in the correlation between free asparagine, nitrogen, and the nitrogen to sulphur ratio in wheat grain (Table 1), and implies that wheat uses free asparagine as a nitrogen store in the grain when sulphur is limiting (reviewed in [8]). Application of more sulphur is, therefore, desirable for both traits, except for its environmental pollution effects [43], whereas a balance between higher protein/higher free asparagine and lower protein/lower free asparagine must be struck when it comes to nitrogen application. Similar trade-offs arise because of the association of nitrogen with desirable agronomic traits, but there may be solutions in breeding, as discussed below. In the meantime, our advice is that nitrogen application should be accompanied with sufficient sulphur (typically 20 kg sulphur per hectare) to prevent the nitrogen ending up as free asparagine instead of protein.

### 2.2. Free Asparagine and Agronomic Traits

As a result of the positive association between free asparagine and nitrogen application, it might be expected that there would be a similar association between free asparagine and traits related to growth because of the positive relationship between plant growth and nitrogen. Positive correlations between free asparagine and yield have indeed been found (Table 3) but, perhaps surprisingly, these correlations have not been consistent across studies. Xie et al. [44], for example, found that free asparagine (measured in milligrams per gram of protein) was negatively correlated with grain yield in one year when the yield was low (between two and four tonnes per hectare), but positively associated in another year, when the yield was higher (between four and eight tonnes per hectare), suggesting a non-linear relationship. A reduction in plant stress could explain the negative correlation observed over lower yield values, whilst the positive correlation could be due to greater nitrogen availability in the soil. However, the authors note that the relationship between absolute free asparagine content (measured without normalisation to protein) and yield was not as strong as the relationship when the normalisation of free asparagine to protein was performed. The lack of comprehensive yield/free asparagine studies does not provide strong support for hypotheses linking the two traits, but it could be worthwhile investigating the nature of the relationship between free asparagine and yield in more detail in future studies. 

Another interesting correlation shown in Table 3 is that between the asparagine response (measured as the ratio of asparagine in treated vs. asparagine in untreated plants) and the yield gap-based measure of drought tolerance (YDT), as studied by Yadav et al. [45]. YDT provides a measurement of how well a variety performs under drought stress relative to unstressed conditions. The negative correlation between the asparagine response and YDT in the study indicated that plants that were less tolerant to drought tended to accumulate more asparagine. This relationship is consistent with the general observation that free asparagine accumulates under stress (reviewed in [42]), and Yadav et al. [45] suggested that the relationship could be caused by the remobilisation of nitrogen during stress-induced senescence. Curtis et al. [46] also showed that asparagine metabolism is affected by drought stress in wheat, by constructing a detailed network describing the genes and other factors involved, using a Unique Network Identification Pipeline to show the inter-relationships between genes that changed in expression in response to drought stress, in both leaves and roots. 

The relationship between free asparagine and senescence in wheat is not well understood, but Emebiri [20] did find a negative correlation between asparagine and flowering time (Table 3), which may reflect an association between senescence and asparagine. Senescence is known to cause the remobilisation of nitrogen via asparagine and the activation of asparagine synthetases in other species, including sunflower, tobacco, and barley [31,47,48,49], and early senescing barley lines show greater expression of asparagine synthetase in senescing tissues relative to later senescing lines [50]. Navrotskyi et al. [35] also found a positive correlation between free asparagine and the number of days until harvest (Table 3), again implying that longer periods of senescence might be responsible for this association.

**Table 3:** Associations between free asparagine and agronomic measurements.

Asparagine Measure	Agronomic Measure	*r*	*p*	Reference
Log*_e_* back-transformed	Flowering time	−0.67	<0.001	[20]
Untransformed	Plant height	0.41	<0.001	[29]
	TKW	0.03	0.75	
	Mean kernel diameter	0.13	0.11	
	Mean kernel weight	0.06	0.45	
	Yield	−0.14	0.09	
	Precipitation (HH)	−0.85	<0.05	
	Temperature (HH)	0.74	0.10	
Log*_e_* transformation	HLW	−0.40	<0.001	[34]
Untransformed	Mean kernel diameter	0.37	<0.05	[35]
	Mean kernel weight	0.37	<0.05	
	Yield	−0.32	>0.05	
	Days to harvest	0.61	<0.001	
Log_10_ back transformed	TKW	−0.24	<0.01	[23]
	HLW	−0.21	<0.01	
Untransformed	Nitrogen application	0.63	NA	[40]
Untransformed	TKW	−0.27	0.15	[28]
	HLW	−0.07	0.71	
Log*_e_* transformed responses	YDT	−0.73	<0.05	[45]
Per unit protein	Yield (2018)	0.74	NA	[44]
	Yield (2019)	−0.56	NA	
Untransformed	Yield	0.75	<0.001	[51]

TKW (thousand kernel weight), HH (heading to harvest date), HLW (hectolitre weight), YDT (yield gap-based drought tolerance).

Further research in this area could be greatly facilitated by investigating free asparagine accumulation in stay-green varieties of wheat. These varieties show delayed senescence, leading to a prolonged green phenotype, and generally have higher yields and better stress tolerance, although this leads to a trade-off with protein and micronutrient content due to the yield dilution effect [52]. Heyneke et al. [53] undertook an experiment comparing the leaf metabolome of early and late senescing wheat lines and found that asparagine content in the leaf decreased as senescence progressed, but not significantly. The ratio of asparagine to aspartic acid (as well as the ratio of glutamine to glutamic acid) did increase significantly, though, in both early and late senescing lines, as senescence progressed. The authors of this study interpret the increase in nitrogen-rich amino acids (asparagine and glutamine) relative to their precursors (aspartic acid and glutamic acid, respectively) as being indicative of nitrogen remobilisation to other active organs. The remobilisation of free asparagine from senescing leaves to developing grain may, therefore, be a mechanism which connects senescence and grain-free asparagine content. Further investigation of early and late senescing lines should be undertaken to shed more light on the relationship between free asparagine and senescence.

The development of stay-green varieties is part of a larger effort to develop varieties with better nitrogen-use efficiency (NUE), in order to reduce agricultural inputs, since nitrogen fertilisers are a major source of environmental pollution [54]. NUE can be defined in many different ways but is commonly described as a productivity index measuring yield per unit of nitrogen (see [55] for review). Strategies to enhance NUE in wheat may impact grain asparagine content due to effects on nitrogen uptake and partitioning within the plant, especially those methods that modulate genes involved in amino acid synthesis and transport.

For example, Wan et al. [56] reported that overexpression of the starchy endosperm amino acid transporter, *TaAAP13*, in endosperm tissue increased grain size and grain weight, but decreased grain yield and seed number per plant overall, as well as increasing free asparagine content. In another study, Tiong et al. [57] transformed rice, wheat, and barley plants with a stress-inducible barley alanine aminotransferase, OsAnt1:HvAlaAT, resulting in increased grain yield for some of the resulting lines. The authors also showed that asparagine content in the roots and shoots of the mutant rice plants was decreased relative to wild-type plants, but these measurements were not repeated in the mutant wheat plants and grain asparagine content was not measured. Hu et al. [58] also improved NUE using modulated amino acid transporters, but this time by overexpressing an isoform of glutamine synthetase 2. This increased grain yield under conditions of both high and low nitrogen, but total amino acid and glutamine content (with which asparagine is often strongly positively correlated) only increased significantly under high nitrogen conditions. These examples show that the many different strategies for improving NUE are likely to have different effects on grain asparagine content, based on their individual mechanisms, and in combination with different environments and management practices. It is, therefore, important that effects on free asparagine concentration (and, therefore, acrylamide-forming potential) are assessed in plants in which NUE has been improved.

**Figure 2:**
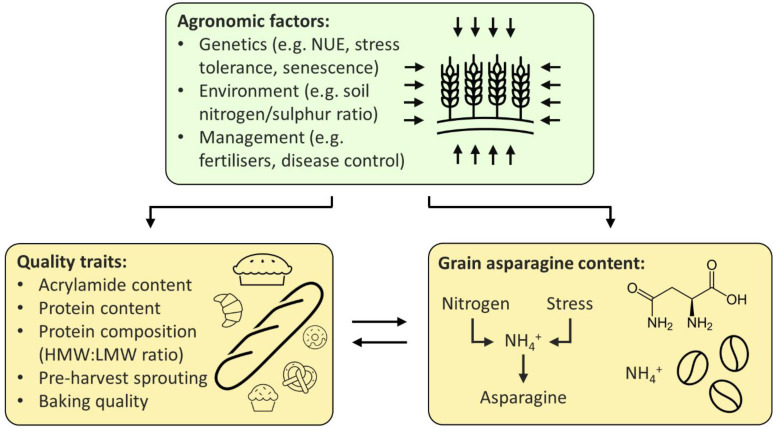
Proposed relationship between agronomic factors, quality traits, and grain asparagine content. Agronomic factors influence both quality traits and grain asparagine content, whilst quality traits and grain asparagine are linked to one another. NUE (nitrogen-use efficiency), HMW (high molecular weight), LMW (low molecular weight).

The relationship between free asparagine, quality, and agronomic traits, as described above, is summarised in Figure 2, below.

## 3. Breeding Wheat with Low Free Asparagine

Selection for desirable traits in wheat (e.g., disease resistance and increased yield) has occurred since humans first started cultivating diploid and tetraploid wheats, approximately 10,000 years ago [59,60]. A historical analysis of varieties, registered from the late 1800s to the present day, indicates that commercial plant breeding has altered the amino acid composition of wheat grain, along with many other agronomic and quality traits [61]. However, free asparagine showed no discernible change across the measured period and another study by Rapp et al. [23] also did not find any temporal trend in free asparagine content across the varieties screened in that study. This is in contrast with Corol et al. [29], who detected a weak negative correlation between variety release year and grain asparagine content (*r* = −0.255, *p* = 0.0019). This slight negative correlation may be due to the decreasing protein content of varieties, as a result of selection for increasing yields and the yield-dilution effect.

The lack of any strong correlation between variety release year and grain asparagine content reflects that free asparagine concentration is not strongly linked to any other traits that have been selected for, over the course of commercial wheat breeding history. However, free asparagine concentration does display a moderate heritability in some studies (Table 4), with the study that used one of the more robust estimates of heritability (Piepho and Möhring method), estimating heritability at 0.65, similar to the heritability estimates obtained for protein content and falling number from the same study [23]. Further accurate measurements of grain asparagine heritability are required to corroborate this, as well as measurements taken across multiple environments, but this indicates that there is scope for reducing the free asparagine content of wheat grain through breeding. However, there is undoubtedly a substantial environmental (E), as well as genetic (G), effect on free asparagine concentration, together with a G × E interaction, which may have discouraged breeders from attempting to develop low-asparagine varieties to date.

**Figure 3:**
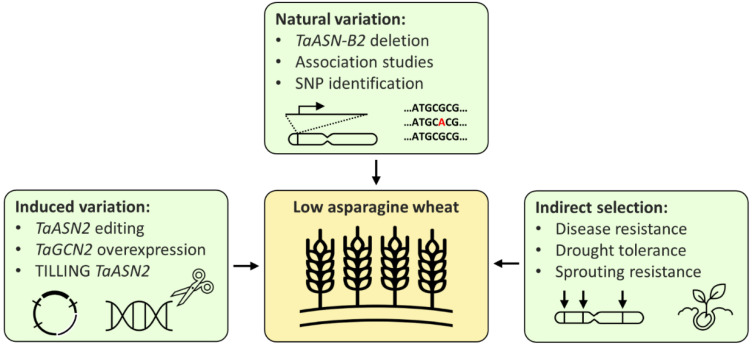
Strategies for the breeding of low-asparagine wheat.

Breeding low-asparagine wheat could potentially be achieved in three main ways: directly, by using either existing or induced variation, or indirectly, through selection for related traits (Figure 3). New wheat varieties are commonly developed using existing variation; however, the only multi-environment quantitative trait locus (QTL) for low-asparagine known at present is the one in which the *TaASN-B2* gene is either present or deleted, which has been shown to affect the free asparagine content of grain in two different field trials [26]. Selection for the *TaASN-B2* deletion represents an easy gain for breeders, but further trials testing the effect of the deletion should be performed to confirm the stability of the effect across more environments. Other QTL controlling grain asparagine content have also been identified, but these have not yet been verified across more than one environment [20,23]. Identification of multi-environment QTL, in combination with genomic and marker assisted selection [23], could enable low-asparagine wheat to be developed, without the time-consuming or expensive need to screen large numbers of plants for asparagine concentration.

Relying on natural variation is limited by the availability of existing variation, whereas techniques that induce or increase variation in the wheat genome could generate new variants with free asparagine content below the normal range. This has been demonstrated by the use of CRISPR/Cas9 technology to ‘knock out’ the *TaASN2* genes, reducing grain asparagine content by up to 90% in glasshouse experiments [25]. The edited lines still need to undergo trials to confirm the stability of this phenotype in the field, but the stability of the ‘natural’ *TaASN-B2* deletion phenotype under field conditions [26] is encouraging, suggesting that the *TaASN2*-edited phenotypes may be similarly stable. However, the interaction between the *TaASN-B2* deletion and sulphur deficiency implies that *TaASN2* variants may not be sufficient to control grain asparagine content during sulphur deficiency or other stresses, again highlighting the effects of E and G × E. On the other hand, the varieties carrying the *TaASN-B2* deletion have intact *TaASN-A2* and *TaASN-D2* genes, whereas the edited lines lack any functional *TaASN2* genes, so the edited lines will be valuable for investigating whether this prevents free asparagine accumulation under conditions of sulphur deficiency or other stresses.

The benefits of inducing variation in candidate genes was also recently demonstrated in a preprint by Alarcón-Reverte et al. [62], in which wheat plants possessing EMS-induced null *TaASN-A2* alleles were grown in the field and tested for grain-free asparagine content. Reductions of between 9% and 34% were achieved, without any negative side effects on quality traits, demonstrating again the utility of induced variation and the lack of strong associations between free asparagine and quality traits.

As a result of the potential loss or partial loss of the low-asparagine phenotype of *TaASN2* knockouts under stress, a third, complementary option for controlling grain asparagine content can also be adopted: breeding for stress tolerance. As discussed above, stress and grain asparagine content are closely linked, and it is often during stress that the highest grain asparagine contents are observed [39,63]. Breeding for stress tolerance could, therefore, ensure that a low-asparagine phenotype would be retained under stress. Selection for other related traits, such as those discussed above (e.g., PHS resistance, delayed senescence), could also provide indirect selection for lower-grain asparagine, but these traits are not as clearly linked with asparagine as asparagine is with stress.

## 4. Conclusions

Asparagine is, of course, an important plant metabolite, and since the discovery that it can be converted to acrylamide during the cooking and processing of food, there has been debate over how much its concentration could be reduced before effects were seen on other important traits. It was also recognized by the food industry that the production of fried, roasted, toasted and baked coffee, potato and cereal products containing no acrylamide at all was not possible, and that they should aim to reduce acrylamide to levels ‘as low as reasonably achievable’ [64]. More recently, at least in the European Union, it has become a regulatory compliance issue, with manufacturers striving to keep the acrylamide levels in their products below benchmark levels set by the European Commission [65]. The European Commission is currently considering replacing benchmark levels (described as ‘performance indicators’) for some products with maximum levels; i.e., levels of acrylamide above which it would be illegal to sell a product [66]. If maximum levels were set at or lower than the current benchmark levels, it would have serious implications for the food industry. There is a paucity of data in the public domain on acrylamide levels in cereal products, but a recent study in Spain found that 15% of breakfast cereals contained acrylamide above the benchmark level, which for wheat-based breakfast cereals is 300 parts per billion (ppb) [67]. Manufacturers could, therefore, face the prospect of product recalls and even prosecution if a maximum level of 300 ppb was imposed. This makes it more important than ever that wheat breeders engage on the acrylamide issue, especially as many strategies involving agronomy and food processing technology have already been implemented [64] and the opportunities for further gains involving those approaches may be limited.

The strategies outlined here show that the breeding of low-asparagine wheat, using natural and induced variation, is feasible and unlikely to negatively impact other traits, with the exception of germination, which may be affected, but only if free asparagine concentration is reduced to very low levels [25]. Furthermore, breeding solutions stand to be more sustainable, cost-effective, and less impactful on flavour than the solutions provided by agronomic and food sciences, and could make additional agronomic or food industry modifications unnecessary. Consequently, development of low-asparagine phenotypes in elite wheat varieties should be considered in future wheat breeding programs.

## Figures and Tables

**Table 4:** Heritability estimates of asparagine in wheat (given to 2 significant figures).

Heritability Method	*h* ^2^	Reference
Broad-sense	0.31	[20]
Surrogate method	0.13	[29]
Piepho and Möhring	0.65	[23]
Broad-sense	0.41	[61]

## Abbreviations

Asn (asparagine), G × E (genotype-by-environment interaction) PHS (pre-harvest sprouting), GWAS (genome-wide association study), QTL (quantitative trait locus/loci), RNAseq (RNA sequencing), HFN (Hagberg falling number), Z-SDS (Zeleny sedimentation index), HMW (high molecular weight), LMW (low molecular weight), *r_p_* (phenotypic correlation), *r_g_* (genotypic correlation), TKW (thousand kernel weight), HH (heading to harvest date), HLW (hectolitre weight), YDT (yield gap-based drought tolerance), nitrogen-use efficiency (NUE), YDT (yield gap-based drought tolerance).
